# Maternal Salivary Glutamate in Women Undergoing Vaginal Delivery: A Comparison Between Epidural Labor Analgesia and Systemic Morphine Analgesia

**DOI:** 10.3390/life16071085

**Published:** 2026-06-28

**Authors:** Mohammad Al Hazaymeh, Omar F. Altal, Atef F. Hulliel, Rami K. Jadallah, Ahmed H. Al Sharie, Dana Saleh, Zaina Giabatti, Omar Hazaymeh, Ashraf Al-Issa, Anas Alrusan, Diab Bani Hani, Ala”a Alhowary

**Affiliations:** 1 Department of Anesthesia, Faculty of Medicine, Jordan University of Science and Technology, Irbid 21110, Jordan; 2 Department of Obstetrics and Gynecology, Faculty of Medicine, Jordan University of Science and Technology, Irbid 21110, Jordan; 3 Faculty of Medicine, Jordan University of Science and Technology, Irbid 21110, Jordan; 4 Department of Medicine, University of Tennessee, Knoxville, TN 37920, USA; 5 Department of Internal Medicine, Southeast Health, Dothan, AL 36301, USA; 6 Faculty of Medicine, An-Najah National University, Nablus P.O. Box 7, Palestine; 7 Department of Anesthesia, Royal Medical Service, Amman 11831, Jordan; 8 Department of Anesthesia, Princess Alexandra Hospital, NHS Trust, Harlow CM20 1QX, UK

**Keywords:** glutamate, epidural, opioid, normal vaginal delivery, enzymatic colorimetric assay, labor analgesia

## Abstract

Introduction: Labor pain is among the most intense forms of acute pain, mediated in part by excitatory glutamatergic neurotransmission within central nociceptive pathways. Glutamate plays a key role in spinal dorsal horn signaling and central sensitization, yet its peripheral dynamics during labor and in response to different analgesic modalities remain unclear. This exploratory study aimed to evaluate whether maternal salivary glutamate levels differ between epidural labor analgesia and systemic morphine analgesia during normal vaginal delivery. Method: In this observational comparative study, 36 women were selected to either epidural analgesia (*n* = 16) or systemic morphine analgesia (*n* = 20). Salivary samples were collected during active labor and analyzed for glutamate concentration using a validated enzymatic colorimetric assay. Clinical and demographic data were recorded. Non-parametric tests were applied due to non-normal distribution of glutamate levels. Results: Baseline maternal and perinatal characteristics were comparable between groups. Median salivary glutamate levels were higher in the epidural group than in the morphine group (5.32 nmol/µL [IQR 2.83–8.00] vs. 3.99 nmol/µL [IQR 2.26–8.03]), but the difference was not statistically significant (*p* = 0.599). Glutamate concentrations showed marked inter-individual variability (0.14–29.89 nmol/µL) and a right-skewed distribution. No significant associations were observed between glutamate levels and maternal age, Body Mass Index, gestational age, birth weight, or obstetric comorbidities. Conclusion: In this exploratory cohort, maternal salivary glutamate concentrations did not differ significantly between epidural labor analgesia and systemic morphine analgesia during labor. The variability observed suggests complex and heterogeneous regulation of peripheral glutamatergic activity in parturition. Further larger-scale studies integrating central and peripheral measurements are warranted.

## 1. Introduction

Labor pain is widely recognized as one of the most intense forms of acute pain experienced by humans [1]. Uterine contractions during the active phase of labor generate visceral nociceptive input transmitted via afferent nerve fibers innervating the uterus, cervix, and lower uterine segment, leading to progressively intensifying pain that is further amplified by somatic pain arising from perineal distension in the second stage [2]. Understanding the neurochemical underpinnings of this pain is fundamental to improving analgesic strategies and optimizing maternal and neonatal outcomes.

Glutamate, the predominant excitatory neurotransmitter in the mammalian central nervous system (CNS), plays a critical role in nociceptive processing [3]. It is released from the central terminals of primary afferent nociceptors into the spinal cord dorsal horn, where it activates both ionotropic receptors, including N-methyl-D-aspartate (NMDA), alpha-amino-3-hydroxy-5-methyl-4-isoxazolepropionic acid (AMPA), and kainate receptors, and metabotropic glutamate receptors (mGluRs) [4,5].

Activation of these receptor systems mediates fast excitatory postsynaptic potentials under physiological conditions, but under conditions of sustained or intense nociceptive stimulation, excess glutamatergic signaling drives the phenomenon of central sensitization, a state of heightened neuronal excitability that underlies hyperalgesia and allodynia [6,7].

A substantial body of preclinical and clinical evidence supports a pivotal role of glutamate and its receptor systems in mediating nociceptive transmission and sustaining central sensitization underlying both acute and chronic pain states [8].

Evidence further suggests that glutamatergic signaling may also be involved in the neurobiology of labor pain, as elevated cerebrospinal fluid concentrations of glutamate and aspartate have been reported in women during active labor compared with non-laboring cesarean controls, supporting a potential contribution of excitatory amino acid neurotransmission to parturition-related nociceptive processing [9].

The two most widely used pharmacological modalities for labor analgesia are epidural analgesia and opioid analgesia [10,11]. Epidural analgesia, which involves the delivery of local anesthetics with or without opioids into the epidural space, provides superior pain relief compared with parenteral opioids and is associated with higher maternal satisfaction [11]. Epidural analgesia is the most effective form of labor pain relief and is used in approximately 30% of laboring women in the United Kingdom and up to 60% in the United States [10]. Systemic opioids, including tramadol and morphine, remain widely used in settings where epidural analgesia is unavailable or declined, offering moderate analgesia with well-characterized maternal and neonatal side effect profiles [12].

This observational study aims to investigate whether maternal salivary glutamate concentrations differ between women receiving epidural analgesia and those receiving systemic morphine analgesia during normal vaginal delivery, in order to better understand the potential relationship between analgesic modality and systemic glutamatergic activity during labor.

## 2. Materials and Methods

### 2.1. Study Design and Participants

A prospective observational, exploratory comparative design was conducted. Participants were recruited and allocated to either the epidural labor analgesia or systemic morphine groups based on the patients’ preference for anesthesia. Both procedures are routinely performed at our center, and no additional medications or interventions were administered for the purpose of this study.

Women eligible for inclusion were those scheduled to undergo normal vaginal delivery (NVD). Inclusion criteria comprised age ≥18 years, American Society of Anesthesiologists (ASA) physical status II, and preoperative fasting time between 8 and 12 h.

### 2.2. Study Setting

The study was conducted under the supervision of one consultant anesthesiologist and one consultant obstetrician. In the delivery room, two intravenous access lines were established for each participant. Standard monitoring, including non-invasive blood pressure, three-lead electrocardiography, and pulse oximetry, was continuously applied throughout labor and the immediate postpartum period.

### 2.3. Epidural Labor Analgesia

Epidural analgesia was administered by a consultant anesthesiologist and supervised senior residents in the labor ward. Two intravenous cannulas were inserted, and standard maternal monitoring (blood pressure, electrocardiogram, and oxygen saturation) was applied throughout the procedure.

Under strict aseptic conditions and after local anesthetic infiltration, the epidural space was identified using the loss-of-resistance technique, and the catheter was inserted at the L2–L3, L3–L4, or L4–L5 interspace when cervical dilation was ≥4 cm. Following confirmation of correct placement, epidural analgesia was initiated with bupivacaine 0.1–0.125% combined with fentanyl 50–100 μg as an initial bolus.

This was followed by patient-controlled epidural analgesia (PCEA) using bupivacaine 0.0625% with fentanyl 2 μg/mL. The infusion rate was maintained at 8–10 mL/h, with a demand bolus of 5 mL and a lockout interval of 5–10 min. Top-up doses were administered as clinically indicated to maintain adequate analgesia, and the infusion was continued until completion of perineal suturing.

The time from epidural initiation to saliva sampling was standardized based on predefined labor stages, as described in the Sample Collection section. Maternal blood pressure and fetal heart rate were continuously monitored, and sensory and motor block levels were assessed periodically. The epidural infusion was discontinued and the catheter removed after delivery.

### 2.4. Systemic Morphine Analgesia

In the Morphine group, at a cervical dilation of approximately 4 cm, women received a single subcutaneous injection of 10 mg of morphine. No additional morphine doses were administered during labor.

### 2.5. Obstetrical Management

All deliveries were conducted by a consultant obstetrician. Upon admission to the delivery room, participants underwent Foley catheterization and continuous external fetal monitoring.

During labor, standard intrapartum monitoring was performed according to institutional protocol. After delivery of the neonate, the umbilical cord was doubly clamped and cut. All women received 10 IU intravenous oxytocin bolus followed by 20 IU infusion over 1 h to facilitate uterine contraction.

Intravenous crystalloids (2–3 L), consisting of 0.9% normal saline and Ringer’s lactate, were administered during labor as part of routine hydration and hemodynamic support. Neonates were immediately assessed by pediatricians, including Apgar scoring and routine perinatal examination.

### 2.6. Sample Collection

Unstimulated saliva samples were collected from each participant during normal vaginal delivery (NVD) at three standardized time points: at baseline (defined as 3 cm cervical dilation before administration of analgesia), at full cervical dilation, and immediately after delivery prior to placental expulsion. Samples were collected using sterile collection tubes. All samples were immediately placed on ice and transported to the Aurum Biotech Diagnostic Laboratory (Amman, Jordan). They were then centrifuged at 3000× *g* for 15 min to remove cellular debris. The resulting supernatant was aliquoted and stored at −80 °C until further analysis.

Each sample was coded using serial numbers by a registered nurse to ensure blinding. Group allocation was concealed from both the principal investigator and the technician. For statistical analysis, the mean of the three time-point measurements per participant was used to represent salivary glutamate levels.

### 2.7. Glutamate Quantification

Glutamate concentrations were measured using the Abcam Glutamate Assay Kit (ab83389, Cambridge, UK) following the manufacturer’s protocol. It is an enzymatic colorimetric assay. This assay enables sensitive quantification of free glutamate in biological samples and does not detect glutamate that is incorporated into proteins or peptides. It is based on an enzymatic colorimetric reaction in which glutamate serves as a specific substrate for the enzyme mixture, producing a colorimetric signal proportional to its concentration.

In brief, standards and appropriately diluted saliva samples were added to 96-well plates, followed by the reaction mix containing the enzyme and developer reagents. Plates were incubated at 37 °C for 30 min, and absorbance was measured at 450 nm using a microplate reader. Concentrations were calculated based on a standard curve generated from supplied calibrators.

Samples were initially analyzed without dilution (1:1); for samples with values exceeding the linear range, 1:2 dilutions were applied to ensure that the measurements fell within the assay’s validated detection range. Final concentrations were calculated by applying the appropriate dilution factor, and all results were reported in nmol/µL as provided by the assay output; no unit conversion was performed.

All samples were measured in duplicate, and the mean value was used for statistical analysis. Blank wells and standards were included on each plate, and standard curve performance was verified according to the manufacturer’s acceptance criteria. All assays were performed within a single analytical run under identical experimental conditions; therefore, intra-assay variability was the primary source of analytical variation.

Regarding assay performance, the manufacturer-reported analytical sensitivity range was 1–10 nmol/well. The intra-assay coefficient of variation (CV) was calculated as (SD/mean) × 100, yielding a value of 6.2%. Inter-assay variability was not applicable, as all samples were processed within a single run without separation across different days, operators, or independent assay batches.

### 2.8. Data Extraction

Clinical and demographic data were extracted from electronic medical records, including body mass index (BMI, kg/m^2^), gestational age (weeks), fetal birth weight (kg), gestational diabetes mellitus (GDM), preeclampsia, antenatal steroid use, and neonatal intensive care unit (NICU) admission, as well as the analgesia group (morphine analgesia or epidural analgesia).

Only participants with complete clinical information and available saliva samples suitable for glutamate analysis were included in the final analysis, while individuals with missing clinical data or unavailable saliva samples were excluded.

### 2.9. Statistical Analysis

Data were entered into a spreadsheet and analyzed using IBM SPSS Statistics for Windows, Version 26.0. Categorical variables were expressed as frequencies and percentages. The normality of the distribution of glutamate concentrations was assessed using the Shapiro–Wilk test; because glutamate was not normally distributed, continuous variables were summarized as medians and interquartile ranges (IQRs) and non-parametric methods were applied throughout. Differences in glutamate concentrations between the two analgesia groups were compared using the Mann–Whitney U test. Associations between glutamate concentrations and continuous maternal and perinatal variables (maternal age, BMI, gestational age, and birth weight) were assessed using the Spearman rank-order correlation coefficient (ρ). Categorical variables were compared between groups using Fisher’s exact test, given the small expected cell counts. A two-sided *p*-value < 0.05 was considered statistically significant. Given the modest sample size and the preference-based (non-randomized) allocation, the study was treated as an exploratory pilot, and its findings are regarded as hypothesis-generating rather than confirmatory. This study was reported in accordance with the Strengthening the Reporting of Observational Studies in Epidemiology (STROBE) guidelines.

## 3. Results

### 3.1. Patient Demographics

A total of 36 women were included in the final analysis, of whom 20 (55.6%) received opioid analgesia and 16 (44.4%) received epidural analgesia. Baseline maternal and perinatal characteristics were similar between the two groups. There were no statistically significant differences in body mass index (BMI), gestational age at delivery, or fetal birth weight (all *p* > 0.05). Also, gestational diabetes mellitus (GDM), preeclampsia, antenatal steroid administration, and neonatal intensive care unit (NICU) admission did not differ significantly between groups (all *p* > 0.05). These findings suggest that the two cohorts were well balanced with respect to clinical and demographic variables, supporting the validity of the subsequent comparisons of glutamate concentrations (Table 1)**.**

The cohort comprised 35 singleton pregnancies and a single twin pregnancy (allocated to the morphine group). Regarding gestational age, 8 women (22.2%) delivered preterm (<37 weeks) and 28 (77.8%) delivered at full term (37 to 41 + 6 weeks); no post-term deliveries (≥42 weeks) occurred.

### 3.2. Comparison of Glutamate Levels

**Figure 1:**
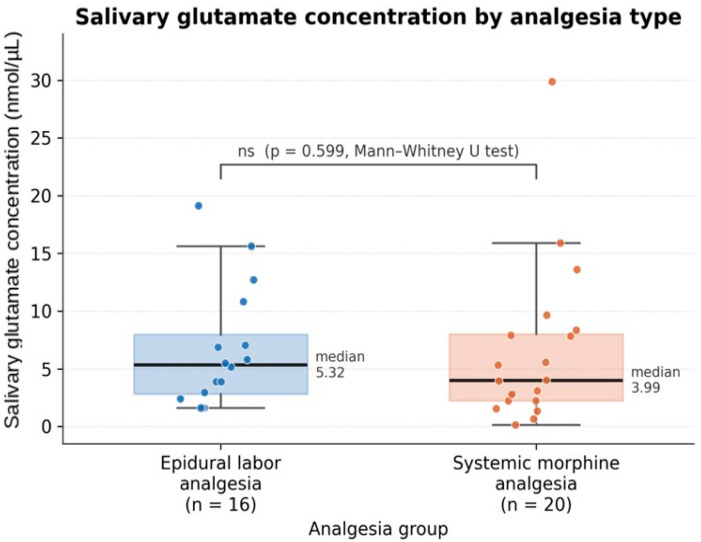
Boxplot of Glutamate levels by analgesia type.

Shapiro–Wilk testing showed that glutamate concentrations were not normally distributed (W = 0.806, *p* < 0.001), and therefore non-parametric analyses were used. Although median glutamate levels were higher in the epidural analgesia group than in the morphine group (5.32 nmol/µL [IQR: 2.83–8.00] vs. 3.99 nmol/µL [IQR: 2.26–8.03], respectively), this difference was not statistically significant (Mann–Whitney U = 143.0, *p* = 0.599). The data exhibited a right-skewed distribution, further justifying the use of non-parametric methods. The distribution of glutamate levels by analgesia type is illustrated in Figure 1.

### 3.3. Secondary Analyses

Secondary analyses were conducted to evaluate the association between glutamate levels and selected maternal and perinatal variables. Given the non-normal distribution of glutamate concentrations, log transformation was considered.

Univariate analyses demonstrated no statistically significant associations between glutamate levels and maternal age (ρ = −0.021, *p* = 0.903), BMI (ρ = −0.040, *p* = 0.857), gestational age (ρ = 0.036, *p* = 0.833), or birth weight (ρ = −0.069, *p* = 0.700). Additionally, no significant difference in glutamate levels was observed based on GDM status (*p* = 0.149) or type of analgesia (*p* = 0.599). These findings suggest that none of the examined variables were significantly associated with glutamate concentrations (Table 2). Salivary glutamate concentrations also did not differ between primigravid (gravidity = 1; *n* = 6; 5.45 [IQR: 2.70–7.01] nmol/µL) and multigravid women (gravidity > 1; *n* = 30; 4.55 [IQR: 2.51–8.24] nmol/µL; Mann–Whitney U = 94.0, *p* = 0.882), nor between nulliparous and parous women (*p* = 0.231). Maternal age, gravidity, and parity were comparable between the two analgesia groups.

When salivary glutamate concentrations were stratified by gestational age category, no significant difference was observed between preterm and full-term deliveries (5.25 [IQR: 2.76–7.26] nmol/µL vs. 3.99 [IQR: 2.26–8.68] nmol/µL; Mann–Whitney U = 109.0, *p* = 0.924). A summary of glutamate concentrations by category is presented in Table 3.

Salivary glutamate concentrations were further examined in relation to a range of maternal and obstetric factors (Table 4). No statistically significant association was found for gestational diabetes mellitus, antenatal steroid use, neonatal intensive care unit admission or mode of admission (elective vs. emergency). Preeclampsia, placenta previa, and instrumental delivery could not be assessed because no such cases were present in the cohort. A nominally higher glutamate concentration was observed in the three women with hypothyroidism (15.63 [IQR: 11.25–15.77] nmol/µL vs. 3.95 [IQR: 2.26–7.85] nmol/µL; *p* = 0.045); however, given the very small subgroup and the large number of comparisons performed, this isolated finding should be regarded as hypothesis-generating only and interpreted with caution.

### 3.4. Distribution of Glutamate Levels

**Figure 2:**
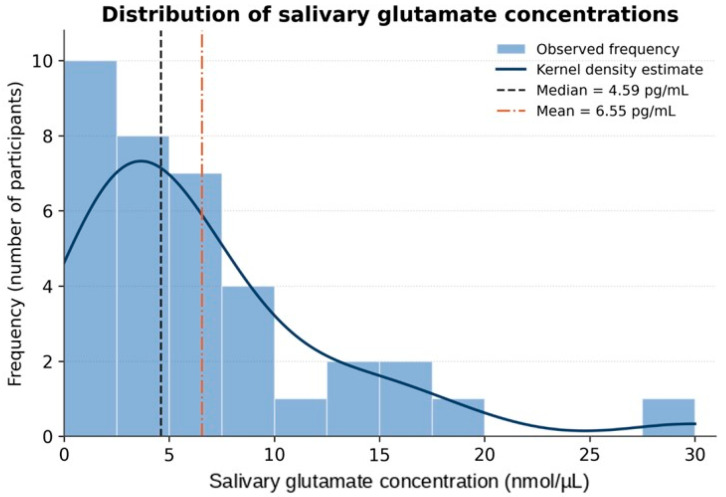
Distribution of glutamate levels (nmol/µL) among the study population.

Glutamate concentrations showed marked inter-individual variability, ranging from 0.14 to 29.89 nmol/µL. The distribution was right-skewed, with the mean (6.55 ± 6.18 nmol/µL) exceeding the median (4.59 nmol/µL). Most observations clustered within the interquartile range of 2.37 to 8.03 nmol/µL, with a long upper tail reflecting higher outlier values. This pattern indicates heterogeneous glutamatergic responses among parturients during labor (Figure 2).

## 4. Discussion

This study investigated whether maternal salivary glutamate concentrations differ between women receiving epidural analgesia and those receiving systemic morphine analgesia during normal vaginal delivery. Our findings revealed that median glutamate levels were numerically higher in the epidural group compared to the morphine group (5.32 nmol/µL vs. 3.99 nmol/µL), but this difference did not reach statistical significance (*p* = 0.599). Additionally, no significant associations were observed between glutamate concentrations and maternal age, BMI, gestational age, birth weight, or gestational diabetes status.

The marked inter-individual variability in glutamate levels, ranging from 0.14 to 29.89 nmol/µL with a right-skewed distribution, underscores the heterogeneous nature of glutamatergic responses during labor.

The absence of a statistically significant difference in glutamate levels between the two analgesic groups may reflect the complex and multifactorial nature of glutamatergic regulation during labor. Glutamate receptors are located in areas of the brain, spinal cord, and periphery involved in pain sensation and transmission [13]. In rodent models, glutamate concentration rises in inflamed tissue [14], and elevated levels of glutamate have been measured in synovial fluid from knee joints of arthritis patients [15]. Central sensitization occurs through the action of glutamate on the NMDA receptor, resulting in increased intracellular calcium levels and kinase activation, leading to hyperalgesia and allodynia [16]. Despite this well-established role in nociceptive processing, differing analgesic modalities during labor did not produce divergent effects on salivary glutamate levels in our cohort.

Epidural analgesia acts by delivering local anesthetics, with or without opioids, into the epidural space to block nociceptive afferent transmission from the uterus and cervix through spinal nerves T10-L1 and sacral segments S2–S4 [10]. Lavand’homme et al. demonstrated that an effective intraoperative neuraxial block of nociceptive inputs contributes to preventing central sensitization [17]. By interrupting the afferent barrage at the spinal level, epidural analgesia may theoretically attenuate glutamate release in the dorsal horn and reduce central sensitization. However, this peripheral blockade may not necessarily translate into detectable changes in systemic salivary glutamate concentrations, as the relationship between central glutamatergic neurotransmission and peripheral levels remains poorly defined.

Conversely, systemic opioids may influence glutamatergic activity through distinct mechanisms. Opioid exposure has been shown to increase release of glutamate from presynaptic terminals within the spinal cord and to enhance NMDA receptor-mediated neuronal responses in dorsal horn neurons [18].

Opioids have been shown to produce sustained activation of NMDA glutamate receptors located on primary afferent nerve terminals, which in turn enhances the transmission of nociceptive signals into the spinal cord [19]. This phenomenon, known as opioid-induced hyperalgesia, involves the activation of the excitatory NMDA receptor and the central glutamatergic system [20]. Thus, opioid analgesia may simultaneously suppress pain perception while promoting glutamatergic excitatory drive, and these opposing effects may contribute to the lack of a clear separation in salivary glutamate between the two analgesic groups.

An important methodological consideration is the use of saliva as the biological medium for glutamate measurement. Jasim et al. (2018) demonstrated a correlation between glutamate concentration in stimulated whole saliva and blood, suggesting a potential relationship between the two fluids [21].

The same group reported that patients with temporomandibular disorder myalgia had significantly higher levels of salivary glutamate compared to pain-free controls, strengthening the importance of salivary glutamate in the pathophysiology of chronic pain conditions [22].

However, Jasim (2023) noted that the salivary levels of pain-related biomarkers, including glutamate, showed significant variation depending on the collection method used [23]. A recent by Zarnegar et al. (2025) reported that salivary glutamate levels fluctuated following experimentally induced acute pain, but the changes were not statistically significant except at a single time point, and the findings did not support its use as a biomarker for acute pain [24]. These findings suggest that while salivary glutamate holds promise as a non-invasive biomarker, its utility in acute pain settings such as labor may be limited by methodological variability and the transient nature of the pain stimulus.

The pronounced inter-individual variability observed in our study is consistent with evidence from pain genetics research. James (2013) noted that there is a high degree of individual variation in pain, very likely due to complex environmental and multiple genetic factors, and that a number of genes play a critical role in determining pain sensitivity and susceptibility to developing chronic pain [25]. Additionally, glutamate levels in the central nervous system are sensitive to ovarian hormone fluctuations, and pregnancy and the postpartum period are associated with the most substantial physiological alterations of female hormones [26]. McEwen et al. (2021) initially observed lower medial prefrontal cortex glutamate levels in healthy pregnant women compared to non-pregnant controls; however, this difference was no longer statistically significant after adjusting for gray matter content [27]. These pregnancy-related neurochemical adaptations may introduce additional variability in peripheral glutamate measurements and could partially explain the heterogeneous glutamatergic responses observed among parturients in our cohort.

The current findings should be interpreted in the context of prior work examining excitatory amino acids during labor. Hsu et al. reported elevated cerebrospinal fluid concentrations of glutamate and aspartate in laboring women compared with non-laboring cesarean controls, supporting a role for excitatory amino acid neurotransmission in parturition-related nociceptive processing [9]. Similarly, Sethuraman et al. reported significantly higher cerebrospinal fluid levels of aspartate, glycine, GABA, and citrulline in women experiencing active labor pain compared to women undergoing cesarean section without labor pain [28].

However, cerebrospinal fluid sampling provides a direct measure of central nervous system neurochemistry, whereas salivary glutamate represents a peripheral compartment that may not faithfully mirror central glutamatergic activity. This compartmental difference is an important consideration when comparing the present findings with earlier cerebrospinal fluid-based studies.

Several limitations of this study merit consideration. First, the relatively small sample size (*n* = 36) and the absent of a true control group limits statistical power and may have been insufficient to detect a modest but clinically meaningful difference in glutamate concentrations between groups. Importantly, the non-significant differences observed do not establish equivalence between the two analgesic modalities, as the study was likely underpowered to detect modest effects. Second, because group allocation was based on patient preference rather than randomization, the groups may differ in unmeasured characteristics, such as pain intensity, anxiety, parity, or cervical dilation at the time of analgesia administration, introducing a risk of selection bias. Consequently, the findings should be interpreted as exploratory and associative rather than causal. Third, pain intensity was not quantified using a visual analog scale or numeric rating scale. Fourth, salivary glutamate concentrations may be influenced by dietary intake, oral microbiome composition, and salivary flow rate, which were not controlled for in this study. Fifth, routinely administered peri-partum medications, including ranitidine, metoclopramide, oxytocin, and intravenous crystalloids, may influence stress, inflammatory, and metabolic pathways as well as salivary composition, representing potential additional confounders. Finally, the absence of a non-analgesic control group limits the ability to determine whether either analgesic modality altered glutamate levels relative to unmedicated labor.

Future studies should consider larger sample sizes with adequate statistical power, serial sampling at multiple time points during labor, and paired analysis of salivary and plasma glutamate concentrations to establish concordance between these compartments in the obstetric population. Inclusion of a non-analgesic control group would help determine whether analgesia modality independently influences glutamate levels. Evaluation of other excitatory and inhibitory amino acid neurotransmitters, including aspartate and GABA, alongside glutamate, may provide a more comprehensive understanding of the neurochemical milieu during labor

## 5. Conclusions

In conclusion, this exploratory study did not detect a statistically significant difference in salivary glutamate concentrations between women receiving epidural labor analgesia and those receiving systemic subcutaneous morphine analgesia during vaginal delivery. Given the small sample size and the preference-based allocation, this absence of a significant difference should not be interpreted as evidence of equivalence between the two modalities. The inter-individual variability observed highlights the complex and multifactorial regulation of peripheral glutamate during parturition. While glutamate remains a central mediator of nociceptive processing, its measurement in saliva as a peripheral biomarker of labor pain requires further validation. These findings contribute to the emerging literature on glutamatergic biomarkers in obstetric pain and provide a foundation for future, adequately powered investigations with standardized sampling, concurrent pain scoring, and multimodal neurochemical assessments.

## Figures and Tables

**Table 1:** Baseline Maternal and Clinical Characteristics by Analgesia Type. Continuous variables are presented as median [interquartile range] and were compared using the Mann–Whitney U test; categorical variables are presented as *n* (%) and were compared using Fisher’s exact test. BMI, body mass index; GDM, gestational diabetes mellitus; NICU, neonatal intensive care unit.

Characteristic	Morphine Analgesia (*n* = 20)	Epidural Analgesia (*n* = 16)	*p*-Value
Maternal age (years)	27.6 [24.7–33.2]	30.1 [25.4–33.3]	0.474
Gravidity	3.0 [2.8–4.2]	2.0 [2.0–3.2]	0.257
Parity	2.0 [1.0–3.0]	1.0 [0.0–2.2]	0.228
BMI (kg/m^2^)	31.0 [27.95–31.24]	27.5 [24.97–29.00]	0.103
Gestational age (weeks)	38.0 [37.1–39.4]	39.3 [37.1–39.6]	0.690
Fetal birth weight (kg)	3.09 [2.96–3.25]	3.24 [3.00–3.35]	0.376
GDM	1 (5.0%)	0 (0%)	1.000
Preeclampsia	0 (0%)	0 (0%)	1.000
Antenatal steroid use	1 (5.0%)	2 (12.5%)	0.574
NICU admission	1 (5.0%)	3 (18.8%)	0.303

**Table 2:** Univariate associations between Fetal and Maternal variables and Glutamate levels. Maternal age, BMI, gestational weeks, and birth weight were assessed using the Spearman rank-order correlation coefficient (ρ); GDM and anesthesia (type of analgesia) were assessed using the Mann–Whitney U test. ρ, Spearman correlation coefficient; U, Mann–Whitney U statistic; BMI, body mass index; GDM, gestational diabetes mellitus.

Variable	Statistic	*p*-Value
Maternal age	ρ = −0.021	0.903
BMI	ρ = −0.040	0.857
Gestational weeks	ρ = 0.036	0.833
Birth weight	ρ = −0.069	0.700
GDM	U = 2.0	0.149
Anesthesia	U = 143.0	0.599

**Table 3:** Maternal salivary glutamate concentrations stratified by gestational age category. Differences across categories were not statistically significant. No post-term (≥42 weeks) deliveries were recorded in the cohort.

Gestational Age Category	*n*	Glutamate, Mean ± SD (nmol/µL)	Glutamate, Median [IQR] (nmol/µL)
Preterm (<37 weeks)	8	5.51 ± 3.78	5.25 [2.76–7.26]
Full term (37–41 + 6 weeks)	28	6.85 ± 6.73	3.99 [2.26–8.68]

**Table 4:** Maternal salivary glutamate concentrations in relation to maternal and obstetric factors. Binary factors were compared using the Mann–Whitney U test.

Maternal/Obstetric Factor	Comparison	*n*	Glutamate, Median [IQR] (nmol/µL)	*p*-Value
Antenatal steroid use	Yes vs. No	3/33	2.40 vs. 5.34	0.188
NICU admission	Yes vs. No	4/32	6.11 vs. 3.99	0.563
Mode of admission	Emergency vs. Elective	2/34	3.78 vs. 4.69	0.604
Menstrual irregularity (history)	Yes vs. No	1/35	15.91 vs. 4.03	0.149
Any maternal comorbidity	Yes vs. No	7/29	6.87 vs. 3.95	0.194
Hypothyroidism	Yes vs. No	3/33	15.63 vs. 3.95	0.045
Abortions (≥1 vs. 0)	Yes vs. No	12/24	6.60 vs. 3.93	0.275

## Data Availability

The datasets used and/or analyzed during the current study are presented in the tables and text.
